# European trauma guideline compliance assessment: the ETRAUSS study

**DOI:** 10.1186/s13054-015-1092-5

**Published:** 2015-12-08

**Authors:** Sophie Rym Hamada, Tobias Gauss, Jakob Pann, Martin Dünser, Marc Leone, Jacques Duranteau

**Affiliations:** Department of Anaesthesiology & Critical Care, AP-HP, Hôpital Bicêtre, Hôpitaux Universitaires Paris Sud, 78 rue du Général Leclerc, 94275 Le Kremlin Bicêtre, France; Department of Anaesthesiology & Critical Care, AP-HP, Hôpital Beaujon, Hôpitaux Universitaires Paris Nord Val de Seine, 100 boulevard du Général Leclerc, 92110 Clichy, France; Department of Anaesthesiology, Perioperative and General Intensive Care Medicine, Salzburg University Hospital and Paracelsus Private Medical University, Müllner Hauptstrasse 48, 5020 Salzburg, Austria; Department of Anaesthesiology & Critical Care, Hôpital Nord, Assistance Publique-Hôpitaux de Marseille, Aix Marseille Université, Marseille, France

## Abstract

**Introduction:**

Haemorrhagic shock is the leading cause of preventable death in trauma patients. The 2013 European trauma guidelines emphasise a comprehensive, multidisciplinary, protocol-based approach to trauma care. The aim of the present Europe-wide survey was to compare 2015 practice with the 2013 guidelines.

**Methods:**

A group of members of the Trauma and Emergency Medicine section of the European Society of Intensive Care Medicine developed a 50-item questionnaire based upon the core recommendations of the 2013 guidelines, employing a multistep approach. The questionnaire covered five fields: care structure and organisation, haemodynamic resuscitation targets, fluid management, transfusion and coagulopathy, and haemorrhage control. The sampling used a two-step approach comprising initial purposive sampling of eminent trauma care providers in each European country, followed by snowball sampling of a maximum number of trauma care providers.

**Results:**

A total of 296 responses were collected, 243 (81 %) from European countries. Those from outside the European Union were excluded from the analysis. Approximately three-fourths (74 %) of responders were working in a designated trauma centre. Blunt trauma predominated, accounting for more than 90 % of trauma cases. Considerable heterogeneity was observed in all five core aspects of trauma care, along with frequent deviations from the 2013 guidelines. Only 92 (38 %) of responders claimed to comply with the recommended systolic blood pressure target, and only 81 (33 %) responded that they complied with the target pressure in patients with traumatic brain injury. Crystalloid use was predominant (n = 209; 86 %), and vasopressor use was frequent (n = 171, 76 %) but remained controversial. Only 160 respondents (66 %) declared that they used tranexamic acid always or often.

**Conclusions:**

This is the first European trauma survey, to our knowledge. Heterogeneity is significant across centres with regard to the clinical protocols for trauma patients and as to locally available resources. Deviations from guidelines are frequent, differ from region to region and are dependent upon specialty training. Further efforts are required to provide consensus guidelines and to improve their implementation across European countries.

**Electronic supplementary material:**

The online version of this article (doi:10.1186/s13054-015-1092-5) contains supplementary material, which is available to authorized users.

## Introduction

Haemorrhagic shock is the leading cause of preventable death in trauma patients [1, 2]. Organisation of care, volume of admissions and implementation of massive haemorrhage protocols can reduce mortality [3, 4]. Increasing compliance with the 2013 European trauma guidelines provides an opportunity to improve clinical care [5]. These guidelines emphasise a comprehensive, multidisciplinary approach to trauma care and underline the need for implementing and adhering to evidence-based management protocols. Nevertheless, educational tools alone may not be sufficient to change clinical practice [6, 7]. Evaluation of clinical practice through surveys may facilitate this change and raise awareness.

The aim of the European Traumatic Shock Survey was to evaluate the current practice of European physicians involved in the acute management of trauma patients with respect to the 2013 guidelines for the management of bleeding and coagulopathy following major trauma.

## Material and methods

### Questionnaire development

The Trauma and Emergency Medicine (TEM) section of the European Society of Intensive Care Medicine (ESICM) designated a working group consisting of physicians involved in trauma care in different European countries. The questionnaire was developed in a five-step process using a nonprobability design that included purposive and snowball sampling [8]. After each step, the working group improved the questionnaire according to the feedback provided. As the survey was based on voluntary participation and information disclosure, the study protocol did not undergo review by an ethics committee. Voluntary participation was taken as consent. Data collection was anonymous.

#### Item generation

First, two members of the working group (SRH, TG) constructed a questionnaire based on central recommendations of the 2013 updated management guidelines [5]. Second, all working group members reviewed the questionnaire. A Delphi method was used for final validation of the questionnaire. Third, 15 independent physicians involved in trauma care in 5 European countries pretested the questionnaire. This was aimed at interpreting the appropriateness of questions in a representative sample. Fourth, a survey service (SurveyMonkey) was used to generate the web interface. Fifth, ten physicians in five European countries evaluated the pilot to assess the layout of the questionnaire.

The questionnaire consisted of 50 questions (Additional file 1) covering the following topics: (1) structural and organisational data regarding hospital and trauma care, (2) haemodynamic resuscitation targets, (3) fluid management, (4) transfusion and coagulopathy management and (5) methods to identify and control bleeding.

#### Sampling

The working group preferentially identified physicians involved in trauma care in Europe (purposive sampling). ESICM national leaders were contacted, and an exhaustive list of representatives from the various scientific societies, associations and foundations involved in trauma care in Europe (emergency medicine, surgery, anaesthesiology and critical care) was created. The authors of studies about trauma care within the last 5 years were screened and contacted. All these potential trauma care representatives were personally solicited via email. They were invited to answer the survey and to spread the information among their peers and/or society members and set up links to the questionnaire on their websites (snowball sampling).

#### Questionnaire dissemination

The questionnaire was published online on the ESICM website from 7 March until 12 June 2014 [9]. A first email was sent to the entire list of identified trauma-related practitioners. After the first-round email, two reminders were sent at 3 weeks and 9 weeks.

The study was endorsed by the European Critical Care Research Network of the ESICM.

### Statistical analysis

Categorical data were assessed and depicted by frequencies (count) and proportions (percentages). Continuous data were expressed as median values with interquartile ranges [1–3] or mean values with standard deviations according to Gaussian distribution. Data were compared using the χ^2^ test (nominal data), Wilcoxon rank test (nonparametric continuous data) or Student’s *t* test (Gaussian continuous data), as appropriate.

The features of guideline compliance were analysed for 13 recommendations independent of the structure of care: existence of a damage control protocol, existence of a massive transfusion protocol, arterial pressure targets (with and without traumatic brain injury (TBI)), use of vasopressors, haemoglobin targets (with and without TBI), transfusion rates and transfusion ratios, use of tranexamic acid, calcium and fibrinogen targets. The mean scores of each subgroup of structural features were compared using analysis of variance to identify the main characteristics of the guidelines.

Statistical analyses were performed using JMP version 8 software (SAS Institute, Cary, NC, USA). Differences were considered significant if the *p* value was <0.05.

## Results

### Structural and organisational data regarding hospital and trauma care

**Fig. 1:**
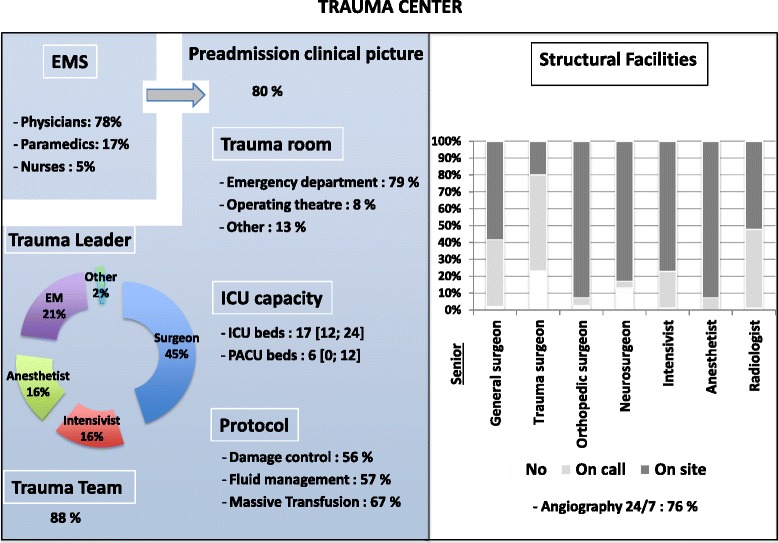
Organisational pattern of trauma centres in Europe (n = 180). *EMS* prehospital Emergency Medical System, *EM* emergency medicine physician, *ICU* intensive care unit, *PACU* Post acute care unit

A total of 296 responses were collected, 243 (81 %) from practitioners in European countries and 53 (19 %) from those in non-European countries. Survey responses from outside the European Union were excluded from the analysis. The geographical distribution of the European responders is shown in Table 1. The characteristics of responders are presented in Table 2. With regard to structural and organisational aspects, 180 responders (74 %) worked in a designated trauma centre. The organisational pattern of European trauma centres according to practitioners’ statements is displayed in Fig. 1.

**Table 1:** Geographical area of practice of the respondents

Country	Number of respondents (%)
France	51 (20 %)
United Kingdom	27 (11 %)
Finland	22 (9 %)
Germany	21 (8.5 %)
Austria	16 (6.5 %)
Spain	16 (6.5 %)
Portugal	13 (5.5 %)
Norway	12 (5 %)
Italy	10 (4 %)
Sweden	9 (3.5 %)
Netherlands	7 (3 %)
Denmark	5 (2 %)
Poland	5 (2 %)
Switzerland	5 (2 %)
Belgium	4 (1.5 %)
Hungary	4 (1.5 %)
Czech Republic	3 (1 %)
Greece	3 (1 %)
Slovakia	3 (1 %)
Lithuania	2 (1 %)
Andorra. Ireland. Latvia. Luxemburg. Romania	1 (0.5 %)^a^
Total	243 (100 %)

^a^ Number of respondents for each country

**Table 2:** Personal and institutional characteristics of the 243 European respondents

	n (%) of respondents
Primary specialty	
Anesthesiology	81 (33 %)
Intensive Care	81 (33 %)
Emergency Medicine	31 (12 %)
Trauma surgery	26 (11 %)
General surgery	19 (7.8 %)
Other	5 (2 %)
Type of ICU	
Mixed (medical & surgical)	172 (71 %)
Surgical & Neurosurgical	41 (17 %)
Surgical	20 (8 %)
Trauma ICU	10 (4 %)
Hospital type	
University affiliated/ teaching	226 (93 %)
Non teaching	12 (5 %)
Other	5 (2 %)
Number of ISS > 15 per Year (Trauma centers)	*n = 180 (74 %)*
< 100	38 (21 %)
100- 200	48 (27 %)
200- 500	56 (31 %)
> 500	13 (7 %)
Do not know	25 (14 %)
Number of ISS > 15 per Year (Non trauma centers)	*n = 63 (26 %)*
< 100	38 (60 %)
100- 200	7 (11 %)
Do not know	18 (28 %)

*ICU* Intensive Care Unit, *ISS* Injury Severity Score

### Haemodynamic resuscitation targets

**Fig. 2:**
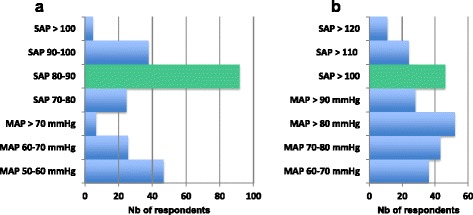
Levels of arterial pressure targeted by the responders. **a** Without traumatic brain injury. **b** With traumatic brain injury. *Green colour* represents recommended arterial pressure targets [5]. SAP systolic arterial pressure, *MAP* mean arterial pressure

Among the 243 responders to this section of the survey, 92 (38 %) claimed to comply with a goal of systolic arterial blood pressure between 80 mmHg and 90 mmHg in patients in haemorrhagic shock without TBI. Thirty-three responders (18 %) and twenty-four responders (10 %) declared targeting higher levels and lower levels, respectively, of systolic arterial pressure. Mean arterial pressure was chosen as a target by 32 responders (34 %). For patients with TBI, responders 80 (33 %) declared that they complied with 2013 guideline recommendations. Also for patients with TBI, 81 responders (33 %) targeted systolic arterial blood pressure (Table 3 and Fig. 2).

**Table 3:** Hemodynamic and fluid management according to respondents specialty

	Total (n = 243)
Monitoring	
HR	212 (87)
Urine output	168 (69)
Lactate clearance	161 (66)
ScVO2	69 (28)
Central VP	64 (36)
Pulse Pressure	60 (25)
Cardiac index	51 (21)
Pressure targets (no TBI)	
SAP 70-80 mmHg	92 (38)
SAP 80-90 mmHg	25 (10)
SAP > 90 mmHg	38 (16)
MAP 50-60 mmHg	47 (20)
MAP 60-70 mmHg	26 (11)
MAP > 70 mmHg	7 (3)
*No answer*	3 (1)
Pressure targets with TBI	
SAP > 100 mmHg	46 (19)
SAP > 110 mmHg	24 (10)
SAP > 120 mmHg	11 (5)
MAP 60-70 mmHg	36 (15)
MAP 70-80 mmHg	43 (18)
MAP ≥ 80 mmHg	52 (22)
MAP ≥ 90 mmHg	28 (12)
*No answer*	3 (1)
Vasopressors	n = 224
Use ( Yes)	171 (76)
> 500 ml	23 (13)
> 1000 ml	73 (43)
> 2000 ml	56 (33)
> 3000 ml	19 (11)
Fluid	
Ringer Lactate	133 (55)
Normal saline	90 (37)
HES	37 (15)
Gelatines	37 (15)
Hypertonic saline	32 (13)
Balanced crystalloids	61 (25)

Percentages are calculated on the total number of respondents (n of each column) except for vasopressor (n of respondents in the line Vasopressor); as “no answer” for vasopressor = 19

*SAP* systolic arterial pressure, *TBI* traumatic brain injury, *MAP* mean arterial pressure, *HR* heart rate

### Fluid management and vasopressor use

With respect to fluid resuscitation before administration of blood products, multiple responses were possible. Regarding the use of balanced solutions, lactated Ringer solution was reported by 173 responders (71 %) and normal saline was used by 91 responders (37 %) ((Table 3 and Additional file 2). Starches, gelatins and hypertonic solutions were administered by 36 (15 %), 36 (15 %) and 32 (13 %) responders, respectively.

With respect to vasopressors, 19 who returned surveys (8 %) did not answer this question. Among responders, 171 (76 %) agreed with their use and 53 (24 %) disagreed. Fifty-eight responders (26 %) considered the use of vasopressors potentially deleterious. Among those employing vasopressors, norepinephrine was the first-line agent used by 169 responders (84 %).

### Transfusion and coagulation management

More than 95 % of responders described complying with the recommended haemoglobin transfusion trigger of 7–9 g/dl. Responders targeted higher haemoglobin levels in patients with TBI than in those without TBI (9 [8–10] g/dl vs 8 [8, 9] g/dl, *p* < 0.05). Haemoglobin targets were higher in trauma centres than in nontrauma centres for all patients, and specifically for both patients without TBI (8 [7–9] g/dl vs 7 [7, 8] g/dl, *p* < 0.05) and patients with TBI (9 [8–10] g/dl vs 8 [8, 9] g/dl, *p* < 0.05). MTPs (massive transfusion protocol) were available in some form in 146 institutions (66 %). MTP initiation was based on clinical and biological data according to 167 responders (83 %). Eleven responders (8 %) indicated using a score to predict transfusion requirements. The recommended ratio of fresh frozen plasma to red blood cells as a minimum of 1:2 was followed by 178 responders (80 %).

For the diagnosis of acute traumatic coagulopathy, 87 responders (35 %) claimed to use viscoelastic methods and 47 (19 %) reported using point-of-care devices. Standard laboratory variables included fibrinogen 161 (66 %), platelets 146 (60 %), prothrombin time 123 (50 %) and partial thromboplastin time [*n* = 108 (44 %)]. The first-line agents used to treat coagulation disorders were stated to be fresh frozen plasma [*n* = 201 (83 %)], platelets [*n* = 187 (77 %)] and fibrinogen concentrate [*n* = 163 (66 %)]. Of note, 121 responders (50 %) reported the use of prothrombin complex concentrates. Activated factor VII, cryoprecipitate and desmopressin use were described by 68 (28 %), 41 (17 %) and 33 (14 %) of responders, respectively. With respect to tranexamic acid, 160 responders declared that they used it always or very often (66 %), 35 sometimes or fairly often (14 %) and 21 almost never or never (7 %). Notably, 27 participants (13 %) did not respond to this question.

### Diagnosis of haemorrhage and haemorrhage control measures

A total of 215 responses were obtained for this section. First-line procedures were chest radiographs and ultrasound for 75 and 126 responders (35 % and 57 %), respectively. For 155 (72 %) responders, computed tomography was the second procedure. The use of peritoneal lavage was the last procedure for 194 responders (90 %).

With respect to haemostatic procedures in patients with pelvic fractures, external compression by a sheet or a pelvic belt in pelvic trauma was considered first-line treatment by 181 responders (84 %). The second-line therapy was interventional radiology [n = 103 (48 %)], Ganz clamp [n = 69 (32 %)] or pelvic packing [n = 62 (29 %)]. Significant variability in these procedures was observed between countries. Interventional radiography was the first procedure used in France and Spain, whereas the Ganz clamp and pelvic packing were the first-line procedures in Germany and the United Kingdom.

### Compliance with guidelines

The results of analysis of guideline compliance characteristics are displayed in Table 4. Working in a trauma centre, specifically in a dedicated trauma intensive care unit (ICU) as an anaesthesiologist or intensivist, was associated with improved guideline compliance rates. In contrast, academic affiliation, ICU size and the specialty of trauma leaders were not linked to better guideline compliance. However, working as an anaesthesiologist or intensivist in a trauma centre or a dedicated trauma ICU was correlated with a higher level of guideline compliance.

**Table 4:** Characteristics of guideline compliance

	Respondent characteristics	Guideline compliance score^a^	*p* Value
Specialty	Anaesthesiology (n = 81)	7.9 ± 1.9	0.004
	Emergency medicine (n = 31)	6.4 ± 2.7	
	Intensive care (n = 81)	7.1 ± 2.7	
	Surgery (n = 46)	6.4 ± 3.2	
ICU type	Exclusively trauma (n = 10)	8.6 ± 2.0	0.03
	Mixed surgical-medical (n = 172)	6.9 ± 2.7	
	Surgical/neurosurgical (n = 61)	7.7 ± 2.3	
Hospital type	University (n = 55)	7.1 ± 2.6	0.80
	Nonuniversity (n = 188)	7.2 ± 2.7	
Trauma centre	Trauma centre (n = 180)	7.4 ± 2.5	0.016
	Nontrauma centre (n = 63)	6.6 ± 2.8	
Trauma leader	Anaesthesiologist (n = 71)	7.6 ± 1.8	0.12
	Emergency medicine (n = 53)	6.6 ± 2.7	
	Intensivist (n = 50)	7.0 ± 2.8	
	Surgeon (n = 104)	7.5 ± 2.7	
ICU beds	<15 beds (n = 130)	7.2 ± 2.4	0.96
	≥15 beds (n = 111)	7.2 ± 2.4	

*ICU* intensive care unit

Data are presented as mean ± standard deviation

^a^Of a possible score of 13, as 13 recommendations were analysed: existence of a damage control protocol, existence of a massive transfusion protocol, arterial pressure targets [in patients with or without traumatic brain injury TBI)], use of vasopressor, haemoglobin targets (in patients with or without TBI), transfusion ratios (plasma to red blood cells, platelets to red blood cells, and platelet numeration transfusion target), use of tranexamic acid, and calcium and fibrinogen targets

## Discussion

To our knowledge, we report the first European survey focusing on trauma management. It provides a snapshot of trauma patient management across European countries. In addition, this article describes the level of agreement with the 2013 European trauma guidelines. The most striking finding is the variability of adherence to recommendations among responders, countries and protocols.

### Trauma organisation and patient volume

In Europe, blunt trauma dominates epidemiology in small centres as well as in larger centres with more than 500 trauma admissions per year. This differs from North American trauma centre data [10]. In Europe, the trauma admission volume per centre and per year seem to be inferior to those reported in North America. In an important study of patients with an Injury Severity Score higher than 15, Nathens et al. showed the positive impact on length of stay and mortality in centres admitting more than 300 trauma patients per year [11]. This number exceeds the volume reported for most trauma centres in our survey (Table 2). Nevertheless, the comparison of European and North American trauma care systems is made difficult by significant organisational divergences in emergency medical systems and even the primary specialties of trauma leaders (Fig. 2). Furthermore, the European trauma centre organisation, staffing and resources in our survey sample were all quite heterogeneous. That said, the core elements of any trauma system—a dedicated trauma team and a designated and identifiable leader—were reported for the majority of centres.

### Compliance with guidelines

Our survey suggests a large variability in compliance with the 2013 guidelines. This is highlighted by the levels of compliance regarding blood pressure targets, vasopressor use, blood product ratios and use of adjunct haemostatic products. Maintaining a target systolic arterial blood pressure of 80–90 mmHg in patients with ongoing haemorrhage is a relatively high-level recommendation (1C). However, only 38 % of responders stated that they target this level of systolic blood pressure in patients without TBI. Some 35 % of responders still use mean arterial pressure as a target. In contrast, the ongoing controversy about vasopressor use results in its being just a grade 2C recommendation. Nevertheless, vasopressor administration prevails according to 75 % of responders. Only 22 % of them reported considering the potential deleterious effects related to vasopressor use.

Similar conclusions can be drawn for the use of fresh frozen plasma (grade 1C) and the ratio of fresh frozen plasma to red blood cells (grade 2C). Until recently [12], no high-level evidence was available to support this strategy. However, a ratio of fresh frozen plasma to red blood cells between 1:2 and 1:1 seemed highly consensual, even though doubts emerged from a recent study about the benefits and effects of using this measure [13].

Adjunctive agent use was highly variable. The administration of tranexamic acid is supported by a large, multicentre, randomised clinical trial (grade 1A). Despite this study, responders seemed not entirely convinced by this recommendation. In contrast, the use of fibrinogen appeared frequent, even though its level of recommendation is low. Our data also suggest increasing use of viscoelastic diagnostic methods to monitor trauma-associated coagulation disorders.

### Potential explanations for noncompliance

The relatively low rate of guideline compliance we found is not surprising compared with the results of other practice survey studies. For example, overall compliance with the Surviving Sepsis Campaign guidelines was around 31 %, even after a large international educational campaign [14]. One study done in a large, multicentre network in France demonstrated a 24 % rate of compliance with high-level practice recommendations [15]. Of note is the large variability of adherence to a specific guideline in this study, ranging from 20 % to 96 %. This observation was corroborated in a recent survey on shock management [16].

Hence, the level of evidence does not seem to be the major criterion associated with guideline adherence. Both national context and specialty training affect compliance rates. For example, crystalloids are a consensual choice for fluid resuscitation. However, therapeutic choices for fluid in intravenous fluid resuscitation differ between anaesthetists, intensivists, surgeons and emergency physicians. Another example is vasopressor use, in which surgeons are less likely to indulge than intensivists or anaesthetists. Some other important determinants are probably associated with knowledge levels [17, 18], attitudes and personal values. Finally, organisational and administrative models of trauma centres may influence compliance with guidelines [19].

In stressful environments such as trauma centres, health care professionals often adopt heuristic decision-making strategies to reduce cognitive load [20, 21]. Trauma care is stressful and characterised by uncertainty, and, as such, gaps between knowledge and routine practice may evolve [22, 23]. In addition, the low level of evidence for most recommendations probably encourages these processes. Nevertheless, in the face of uncertainty, guidelines should be used to facilitate decision-making processes, thereby reducing the burden of uncertainty and anxiety and reassuring practitioners, patients and patients’ relatives.

### Spread of compliance with guidelines

The univariate analysis showed that the structures specifically organised for trauma care (trauma centres, trauma ICUs) were significantly linked to better guideline compliance. Strikingly, this was independent of their academic label. Along the same lines, a previous study underscored the variations in care between different institutions, impacting patient outcomes [24]. Elsewhere, guideline compliance was associated with improved outcomes [25]. The interest in these results is to show that, among European countries, compliance with guidelines seems to be improved among professionals working in dedicated trauma centres. We also found an association between specialty and guideline compliance. The European guidelines are published by the ESICM that is composed of more anaesthesiologists and intensivists than other specialists. This probably impacted the accuracy of responses. We did not compare the differences between countries, for several reasons. The representation was imbalanced among European countries, and combining different countries (e.g., based on their geographic region) might have been similarly prone to bias, as most countries differ in their trauma care organisation and face different local and/or regional constraints.

### Limitations

Our survey has inherent limitations. First, a selection bias cannot be excluded. Given the process of dissemination, it is difficult to provide an estimation of the nonresponder-to-responder ratio. We targeted practitioners working primarily in trauma care, but of course results from this sample cannot be extrapolated to the practice of all physicians in Europe participating in major trauma care. The overrepresentation of academic hospitals and the low recruitment rate in some quite large European countries may also have introduced further bias. As the survey was produced by the ESICM, physicians from Western European countries were represented more than those from other specialities. Furthermore, any self-reported survey is highly prone to bias; thus we could not assess the gap between routine practice and self-perception. As such, guideline compliance in the present study reflects no more than self-perceived and self-reported compliance.

## Conclusions

The TEM survey delivers several key messages. The heterogeneity in trauma care management and resources across European countries is significant. Deviations from guidelines are frequently reported and seem to be related to geographic region and specialty training. Further efforts are required to provide consensus guidelines and to improve their implementation across Europe. Further studies should be done to examine the effect of guidelines and whether compliance results in improved patient outcomes. Guidelines must not suppress innovation, but they may help the physician to deliver high-quality health care. This effort could be facilitated by a common trauma curriculum for all critical trauma care providers and a centralised European trauma registry.

## Key messages

- Trauma care in Europe is heterogeneous.
- Surgeons, intensivists, anaesthesiologists and emergency medicine physicians share trauma care.
- Deviations from guidelines are frequent.

## Additional files

Additional file 1:
**50-item questionnaire.** (DOCX 39 kb) Additional file 2:
**Haemodynamic and fluid management according to respondent specialty (Table** **3** **supplemental work).** Percentages are calculated on the basis of total number of respondents (‘n’ in each column), except for vasopressors (number of respondents in the Vasopressor row) as 19 gave no answer for this section. *SAP* systolic arterial pressure, *TBI* traumatic brain injury, *MAP* mean arterial pressure. (DOCX 107 kb)

## Abbreviations

EM: Emergency medicine physician
EMS: Emergency Medical System
ESICM: European Society of Intensive Care Medicine
ICU: Intensive care unit
ISS: Injury Severity Score
MAP: Mean arterial pressure
MTP: Massive transfusion protocol
PACU: Post acute care unit
SAP: Systolic arterial pressure
TBI: Traumatic brain injury
TEM: Trauma and Emergency Medicine section of the European Society of Intensive Care Medicine
