# Supplementary material for: European trauma guideline compliance assessment: the ETRAUSS study
Source: Crit Care. 2015 Dec 8;19:423. doi: 10.1186/s13054-015-1092-5 (PMC4672560; doi:10.1186/s13054-015-1092-5)
Supplement: Additional file 1: — 50-item questionnaire. (DOCX 39 kb) [file 13054_2015_1092_MOESM1_ESM.docx]

**ETRAUSS- European Traumatic Shock Survey**

**Questionnaire**

**1. BACKGROUND**

**1. 1. In which country do you practice?** (Drop down Menu- single response)

**1. 2. Main Specialty :** (Drop down Menu- single response)

- Intensive Care Medicine

- Anesthesiology

- Emergency medicine

- Trauma surgery

- General surgery

- Neurosurgery

- Other: ? ***Open field***

**1.3. Type of Intensive Care:** (Drop down Menu- single response- single response)

- Exclusively neurosurgical ICU

- Exclusively Surgical ICU

- Surgical and neurosurgical ICU

- Exclusively Trauma ICU

- Mixed ICU (medical and surgical)

**1.4. Number of ICU beds :** Open Field

**Number of PACU beds (Post Acute Care Unit):** Open Field

**1.5. Institution :** (Drop down Menu- single response)

- University Hospital

- University affiliated hospital

- Regional teaching hospital

- Non teaching hospital

- Other

**1.5. Is your hospital a trauma center?** (Drop down Menu- single response – nominal answer)

- Yes

- No

**1.6. Do you have a dedicated trauma team* caring for the severe trauma patient in the emergency department?** (Drop down Menu- single response – nominal answer)

- Yes

- No

* Identified medical and paramedical agents who are operational when a trauma patient is admitted

**1.7 Is there 24h / 7j**

- **A senior intensivist** : On site / On Call (Drop down Menu)
- **A senior general surgeon**: On site / On Call (Drop down Menu)
- **A senior orthopedic surgeon**: On site / On Call (Drop down Menu)
- **A neurosurgeon**: On site / On Call (Drop down Menu)
- **A senior radiologist**: On site / On Call (Drop down Menu)
- **A trauma surgeon**: On site / On Call (Drop down Menu)
- **An anesthetist** : On site / On Call (Drop down Menu)

**1.8. Who is the trauma leader?** (Multiple choice)

- Intensivist

- Anesthesiologist

- Emergency physician

- Surgeon

- Other

**1.9. Approximate number of patients admitted to your hospital for trauma with ISS>15 / year?** (Drop down Menu- single response)

- > 500

- 200-500

- 100-200

- <100

- I don’t know

**1.10 Where is the trauma bay located?** (Drop down Menu- single response)

- ED

- Operating theatre

- Recovery room

- Other: open field

**1.11 Who are the pre-hospital major trauma care providers in your EMS:** (Drop down Menu- multiple response)

- Physicians

- Paramedics

- Nurses

**1.12 What is the mean ISS in your unit?**

- (Open field)

- I don’t know

**1.13 What is the percentage of:**

- Penetrating trauma : (Open field) / I don’t know

- Blunt trauma : (Open field)/ I don’t know

**1.14 Do you get a clinical picture from the pre-hospital providers before the admission of the patient to the trauma bay?** (Drop down Menu- single response)

- Yes

- No

**1.15- Do you have O group (+ or -) blood products directly available in the trauma bay or at the site where the trauma bay is located?** (Drop down Menu- single response)

- Yes

- No

**1.16- Do you dispose of unfrozen FFP in your trauma bay area?** (Drop down Menu- single response)

- Yes

- No

**1.17- Do you have a stock of frozen FFP that you defreeze yourself in the trauma bay area?** (Drop down Menu- single response)

- Yes

- No

**1.18- Is interventional angiography available 24 hours/7 days in your hospital?** (Drop down Menu- single response)

- Yes

- No

**1.19- Do you have a structured and multidisciplinary damage control resuscitation protocol in your unit that is routinely used?** (Drop down Menu- single response)

**-** Yes

- No

**1.20- Do you have a protocol of fluid resuscitation in your department/ hospital?** (Drop down menu- single response)

- Yes

- No

**2. CLINICAL DATA**

**A. Resuscitation Endpoint**

**During ongoing hemorrhage (before bleeding control)**

**A1- Which hemodynamics parameters do you use to guide the fluid resuscitation?** (Multiple choice)

- Arterial blood pressure

- Heart rate

- Cardiac index

- Lactate clearance

- Central Venous Oxygen Saturation (SCVO2)

- Central venous pressure (CVP)

- Delta pulse pressure (Delta PP)

- Urine output

- Other

**During ongoing hemorrhage (before bleeding control) and without brain trauma.**

**A2- What arterial pressure target do you use for blunt trauma resuscitation?** (Drop down Menu- single response)

- Systolic arterial pressure (SAP) > 100 mmHg

- Systolic arterial pressure (SAP) 90-100 mmHg

**-** Systolic arterial pressure (SAP) 80-90 mmHg

- Systolic arterial pressure (SAP) 70-80 mmHg

- Mean arterial pressure (MAP) 50-60 mmHg

- Mean arterial pressure (MAP) 60 – 70 mmHg

- Mean arterial pressure > 70 mmHg

**A3- What are your target pressures in patients with suspected traumatic brain injury and ongoing hemorrhage after blunt trauma?** (Drop down Menu- single response)

- Systolic arterial pressure (SAP) >100 mmHg
- Systolic arterial pressure (SAP) >110 mmHg
- Systolic arterial pressure (SAP) >120 mmHg
- Mean arterial pressure (MAP) 60-70 mmHg
- Mean arterial pressure (MAP) 70-80 mmHg
- Mean arterial pressure (MAP) > 80 mmHg
- Mean arterial pressure (MAP) > 90 mmHg

**A4. Do you use transcranial Doppler to resuscitate patients with suspected traumatic brain injury?**

**-** Yes

- No

**B. Resuscitation Fluid / Drug**

**During ongoing hemorrhage (before bleeding control)**

**B1- If fluid resuscitation fails to restore arterial pressure, do you use vasopressors?** (Drop down Menu- single response)

- Yes

- No

**If Yes**

**B2- If you use a vasopressor, after which average amount of fluid do you start it?** (Drop down Menu- single response)

- >250 mL

- >500 mL

- >1000 mL

- >2000 mL

- >3000 mL

**During ongoing hemorrhage (before bleeding control)**

**B3- Which vasopressor do you use in first intention?** (Drop down Menu- single response)

- Norepinephrine

- Dopamine

- Epinephrine

- Vasopressin

- Phenylephrine

- Other

**During ongoing hemorrhage (before bleeding control)**

**B4- Do you think that vasopressor infusion during ongoing hemorrhage is deleterious?** (Drop down Menu- single response)

- Yes

- No

- It depends

**During ongoing hemorrhage (before bleeding control)**

**B5- Which type of fluid do you use before getting blood products?** (Multiple choice)

- Ringer’s Lactate

- Normal saline

- HES

- Gelatin

- Hypertonic saline

- Balanced crystalloid

- Other

**During ongoing hemorrhage (before bleeding control)**

**B6- Does fluid resuscitation differ between patients with blunt or penetrating torso injuries ?** (Drop down Menu- single response)

- Yes

- No

**C. Transfusion management**

**C1- What is your target Hb during ongoing hemorrhage?** (Drop down Menu- single response)

- > 6 g/dL

- > 7 g/dL

- > 8 g/dL

- > 9 g/dL

- > 10 g/dL

- > 11 g/dL

**C2- If there is an associate brain injury, what is your transfusion trigger to transfuse red blood cells?** (Drop down Menu- single response)

- < 6 g/dL

- < 7 g/dL

- < 8 g/dL

- < 9 g/dL

- < 10 g/dL

- < 11 g/dL

**C3- Do you have a massive transfusion protocol?** (Drop down Menu- single response)

- Yes

- No

**C4- If Yes how is this massive transfusion protocol triggered?** (Drop down Menu- single response)

- The initial value of hemoglobin

- A score

- Clinical data

- Biological data

- Clinical & Biological data

**C5- Do you warm infusions/blood products during massive transfusion?** (Drop down Menu- single response)

- Yes

- No

**C6- What is the optimal FFP/RBC ratio you apply during ongoing hemorrhage?** (Drop down Menu- single response)

- 1 : 1

- 1 : 1,5

- 1 : 2

- 1 : 3

- Depends on coagulopathy monitoring

**C7- Do you defreeze FFP before the patient ‘s admission to the trauma bay?** (Drop down Menu- single response)

- Never

**-** Rarely

- Sometimes

**-** Often

- Always

**C8- What time does it take for you to dispose of unfrozen FFP?** (Drop down Menu- single response)

- 20 min

- > 20 min

**C9- When do you transfuse platelets during ongoing hemorrhage without traumatic brain injury?** (Drop down Menu- single response)

- Platelets < 100 G/L

-Platelets < 80 G / L

- Platelets < 60 G/L

- Platelets < 50 G/L

- Part of the local massive transfusion protocol

- After a number of RBC transfused: **open field**

**C10- What is the optimal platelet (units)/ RBC ratio in massive transfusion?** (Drop down Menu- single response)

- 1 : 1

- 1 : 1,5

- 1 : 2

- 1 : 3

- Depends on patient platelets count

**D. Coagulation management**

**D1- How do you diagnose coagulopathy?** (Multiple choice)

- Clinical

- POC device (CoaguCheck, INRratio, Hemochron)

- TEG / TEM

- PT, from central laboratory

- PTT, from central laboratory

- Fibrinogen

- Platelet count

**D2- To treat coagulopathy, do you use?** (Multiple choice)

- FFP

- Lyophilized FFP

- Platelets

- Fibrinogen concentrates

- Prothrombin complex

- FEIBA (composition of coagulation factors bypassing Factor VIII activity)

- Activated factor VII

- Factor XIII

- Cryoprecipitate

- DDAVP

- Tranexamic acid

**D3- What is your ionized calcium cut-off-level to administer calcium during ongoing hemorrhage?** (Drop down Menu- single response)

- <1,1

- <1

- < 0,9

- < 0,8

- < 0,7

- < 0,6

**D4- Do you routinely give tranexamic acid in traumatic hemorrhagic shock?** (Drop down Menu- single response)

- Always

- Very often

- Fairly often

- Sometimes

- Almost never

- Never

**D5- When do you give fibrinogen?** (Multiple choice)

- After thromboelastography result

- Fibrinogen <1

- Fibrinogen <1.5

- Fibrinogen <2

- Fibrinogen <2.5

- Fibrinogen <3

- Other

**D6- Do you administer buffer solutions in metabolic acidosis pH <7.2 and hemorrhagic traumatic shock?** (Drop down Menu- single response)

- Yes

- No

**D7- Do you routinely measure temperature in patients with hemorrhagic traumatic shock?** (Drop down Menu- single response)

- Yes

- No

**E. Hemorrhage detection/ Control**

**E1- For patients with blunt trauma, give a sequence of complementary techniques you would use in unstable but “stabilized” patients in hemorrhagic shock**

**(Give a sequence number- ordinal response)**

- X-rays (chest, pelvic)

- FAST (abdominal+ pericardium)

- FAST extended to lung and pleural

- Diagnostic Peritoneal lavage

- Angiography

- CT scan

**E2- What is the preferred and routinely used technique of pelvic hemorrhage control in first intention in your hospital? (Give a sequence number- ordinal response)**

- External Compression (sheet or belt)

- Ganz Clamp

- Pelvic tamponade

- Open surgery

- Interventional angiography

**Auxillary:**

**Do you want to leave your email and receive the results of the survey?**
