# Supplementary material for: European trauma guideline compliance assessment: the ETRAUSS study
Source: Crit Care. 2015 Dec 8;19:423. doi: 10.1186/s13054-015-1092-5 (PMC4672560; doi:10.1186/s13054-015-1092-5)
Supplement: Additional file 2: — Haemodynamic and fluid management according to respondent specialty (Table 3 supplemental work). Percentages are calculated on the basis of total number of respondents (‘n’ in each column), except for vasopressors (number of respondents in the Vasopressor row) as 19 gave no answer for this section. SAP systolic arterial pressure, TBI traumatic brain injury, MAP mean arterial pressure. (DOCX 107 kb) [file 13054_2015_1092_MOESM2_ESM.docx]

|  | **Anesthesiology (n=81)** | **Emergency Medicine (n=31)** | **Intensive Care (n=81)** | **Surgery**  **(n=46)** | **Other**  **(n=4)** | **Total**  **(n=243)** |
| --- | --- | --- | --- | --- | --- | --- |
| **Monitoring**  HR  Urine output  Lactate clearance  ScVO2  Central VP  Pulse Pressure  Cardiac index | 72 (89)  58 (72)  63 (78)  28 (35)  22 (27)  27 (33)  17 (21) | 26 (84)  19 (61)  13 (42)  6 (19)  5 (16)  5 (16)  4 (13) | 71 (88)  58 (72)  60 (74)  25 (31)  26 (32)  22 (27)  25 (31) | 39 (85)  30 (65)  22 (48)  10 (22)  10 (22)  6 (13)  5 (11) | 4 (100)  3 (75)  3 (75)  0 (0)  1 (25)  0 (0)  0 (0) | 212 (87)  168 (69)  161 (66)  69 (28)  64 (36)  60 (25)  51 (21) |
| **Pressure targets (no TBI)**  SAP 70-80 mmHg  **SAP 80-90 mmHg**  SAP > 90 mmHg | 10 (12)  **28 (35)**  14 (17) | 4 (13)  **9 (29)**  7 (23) | 7 (9)  **35 (43)**  6 (7) | 3 (7)  **19 (41)**  14 (30) | 0 (0)  **1 (25)**  3 (75) | 92 (38)  **25 (10)**  38 (16) |
| MAP 50-60 mmHg  MAP 60-70 mmHg  MAP > 70 mmHg  *No answer* | 20 (25)  7 (9)  2 (3)  - | 2 (6)  8 (26)  1 (3)  - | 24 (30)  7 (9)  1 (1)  1 (1) | 1 (2)  4 (9)  3 (7)  2 (4) | 0 (0)  0 (0)  0 (0)  - | 47 (20)  26 (11)  7 (3)  3 (1) |
| **Pressure targets with TBI**  SAP > 100 mmHg  SAP > 110 mmHg  SAP > 120 mmHg | 7 (11)  6 (7)  4 (5) | 9 (29)  3 (10)  0 (0) | 10 (12)  10 (12)  6 (7) | 18 (41)  4 (9)  1 (2) | 2 (50)  1 (25)  0 (0) | 46 (19)  24 (10)  11 (5) |
| MAP 60-70 mmHg  MAP 70-80 mmHg  **MAP ≥ 80 mmHg**  MAP ≥ 90 mmHg  *No answer* | 16 (20)  19 (23)  **21 (26)**  8 (10)  - | 3 (10)  5 (16)  **6 (19)**  5 (16)  - | 13 (16)  14 (17)  **18 (22)**  9 (11)  1 (1) | 3 (7)  5 (11)  **7 (15)**  6 (13)  2 (4) | 1 (25)  0 (0)  **0 (0)**  0 (0)  - | 36 (15)  43 (18)  **52 (22)**  28 (12)  3 (1) |
| **Vasopressors**  **Use ( Yes)**  > 500 ml  >1000 ml  > 2000 ml  > 3000 ml | **n= 81**  **65 (80)**  11 (17)  33 (51)  12 (18)  9 (14) | **n=27**  **17 (63)**  2(12)  11 (65)  2 (12)  2 (12) | **n=74**  **63 (85)**  9 (14)  20 (32)  29 (46)  5 (8) | **n=38**  **24 (63)**  1 (4)  8 (33)  13 (54)  2 (8) | **n=4**  **2 (50)**  0 (0)  1 (50)  0 (0)  1 (50) | **n=224**  **171 (76)**  23 (13)  73 (43)  56 (33)  19 (11) |
| **Fluid**  Ringer Lactate  Normal saline  HES  Gelatines  Hypertonic saline  Balanced crystalloids | 45 (56)  26 (32)  13 (16)  20 (25)  13 (16)  30 (37) | 10 (32)  16 (52)  6 (19)  2 (7)  4 (13)  5 (16) | 44 (54)  36 (44)  13 (16)  12 (15)  11 (14)  20 (25) | 31 (67)  11 (24)  4 (9)  3 (7)  4 (9)  6 (13) | 3 (75)  1 (25)  1 (25)  0 (0)  0 (0)  0 (0) | 133 (55)  90 (37)  37 (15)  37 (15)  32 (13)  61 (25) |

Hhh

**Table 3 supplemental**. Hemodynamic and fluid management according to respondents specialty.

Percentages are calculated on the total number of respondents (n of each column) except for vasopressor (n of respondents in the line Vasopressor) as 19 gave no answer for this section.

SAP: systolic arterial pressure; TBI: traumatic brain injury; MAP: mean arterial pressure
